# Age-related reduction of trunk muscle torque and prevalence of trunk sarcopenia in community-dwelling elderly: Validity of a portable trunk muscle torque measurement instrument and its application to a large sample cohort study

**DOI:** 10.1371/journal.pone.0192687

**Published:** 2018-02-22

**Authors:** Eiji Sasaki, Shizuka Sasaki, Daisuke Chiba, Yuji Yamamoto, Atsushi Nawata, Eiichi Tsuda, Shigeyuki Nakaji, Yasuyuki Ishibashi

**Affiliations:** 1 Department of Orthopedic Surgery, Hirosaki University Graduate School of Medicine, Hirosaki, Japan; 2 Department of Social Medicine, Hirosaki University Graduate School of Medicine, Hirosaki, Japan; 3 Medical Engineering Laboratory, ALCARE Co., Ltd., Tokyo, Japan; University of Tennessee Health Science Center College of Graduate Health Sciences, UNITED STATES

## Abstract

Trunk muscle weakness and imbalance are risk factors for postural instability, low back pain, and poor postoperative outcomes. The association between trunk muscle strength and aging is poorly understood, and establishing normal reference values is difficult. We aimed to establish the validity of a novel portable trunk muscle torque measurement instrument (PTMI). We then estimated reference data for healthy young adults and elucidated age-related weakness in trunk muscle strength. Twenty-four university students were enrolled to validate values for PTMI, and 816 volunteers from the general population who were recruited to the Iwaki Health Promotion Project were included to estimate reference data for trunk muscle strength. Trunk flexion and extension torque were measured with PTMI and KinCom, and interclass correlation coefficients (ICC) were estimated to evaluate the reliability of PTMI values. Furthermore, from the young adult reference, the age-related reduction in trunk muscle torque and the prevalence of sarcopenia among age-sex groups were estimated. The ICC in flexion and extension torque were 0.807 (p<0.001) and 0.789 (p<0.001), respectively. The prevalence of sarcopenia increased with age, and the prevalence due to flexion torque was double that of extension torque. Flexion torque decreased significantly after 60 years of age, and extension torque decreased after 70 years of age. In males over age 80, trunk muscle torque decreased to 49.1% in flexion and 63.5% in extension. In females over age 80, trunk muscle torque decreased to 60.7% in flexion and 68.4% in extension. The validity of PTMI was confirmed by correlation with KinCom. PTMI produced reference data for healthy young adults, and demonstrated age-related reduction in trunk muscle torque. Trunk sarcopenia progressed with aging, and the loss of flexion torque began earlier than extension torque. At age 80, trunk muscle torque had decreased 60% compared with healthy young adults.

## Introduction

Trunk stability plays an important role in low back pain, falls, and locomotive ability [1–4]. Particularly in the elderly, weakness and imbalance of trunk muscle strength are strongly correlated with low back pain [5–9], which is the most common complaint in Japan [10], and one that can have a major socioeconomic impact. Furthermore, the number of spine surgeries in older people is increasing in “super-aged” societies where more than 20% of the population is 65 years or older [11]. There have been reports that wounds caused by invasive surgery cause numerous complications that lead to unsatisfactory outcomes [12].

Besides osteoporotic vertebral bone, it must be recognized that sarcopenia of the trunk muscles leads to dysfunction and affects clinical outcomes, including postoperative rehabilitation [13,14]. However, the contribution of age to decreases in trunk muscle strength is unclear, as reference data in young adults, as well as changes associated with age, have not been investigated in large cohort studies using validated instruments.

There are many reasons why there are no available reference data for trunk muscle strength. One is that accurate, reliable, and easily assessable measurement methods have not been validated. Conventional measurement instruments for trunk muscle include KinCom (Chattanooga Instruments, Chattanooga, TN, USA) [15], Cybex (Computer Sports Medicine Inc., Stoughton, USA) [16], and Biodex (Biodex, Inc., Shirley, NY, USA) [17], and they are large and heavy, making them difficult to carry to study sites. Furthermore, reference data that include age, gender, and other confounders have not been estimated and assessed, as large sample sizes are necessary for these statistical analyses. The lack of reference data makes it difficult to judge the accuracy of clinical comparison studies, because age-related changes such as trunk sarcopenia, focal kyphosis, and other chronic medical conditions likely affect clinical outcomes like pain and function. Thus, the objective evaluation of trunk muscle strength and understanding age-related changes provides important information to allow accurate discussions.

**Fig 1:**
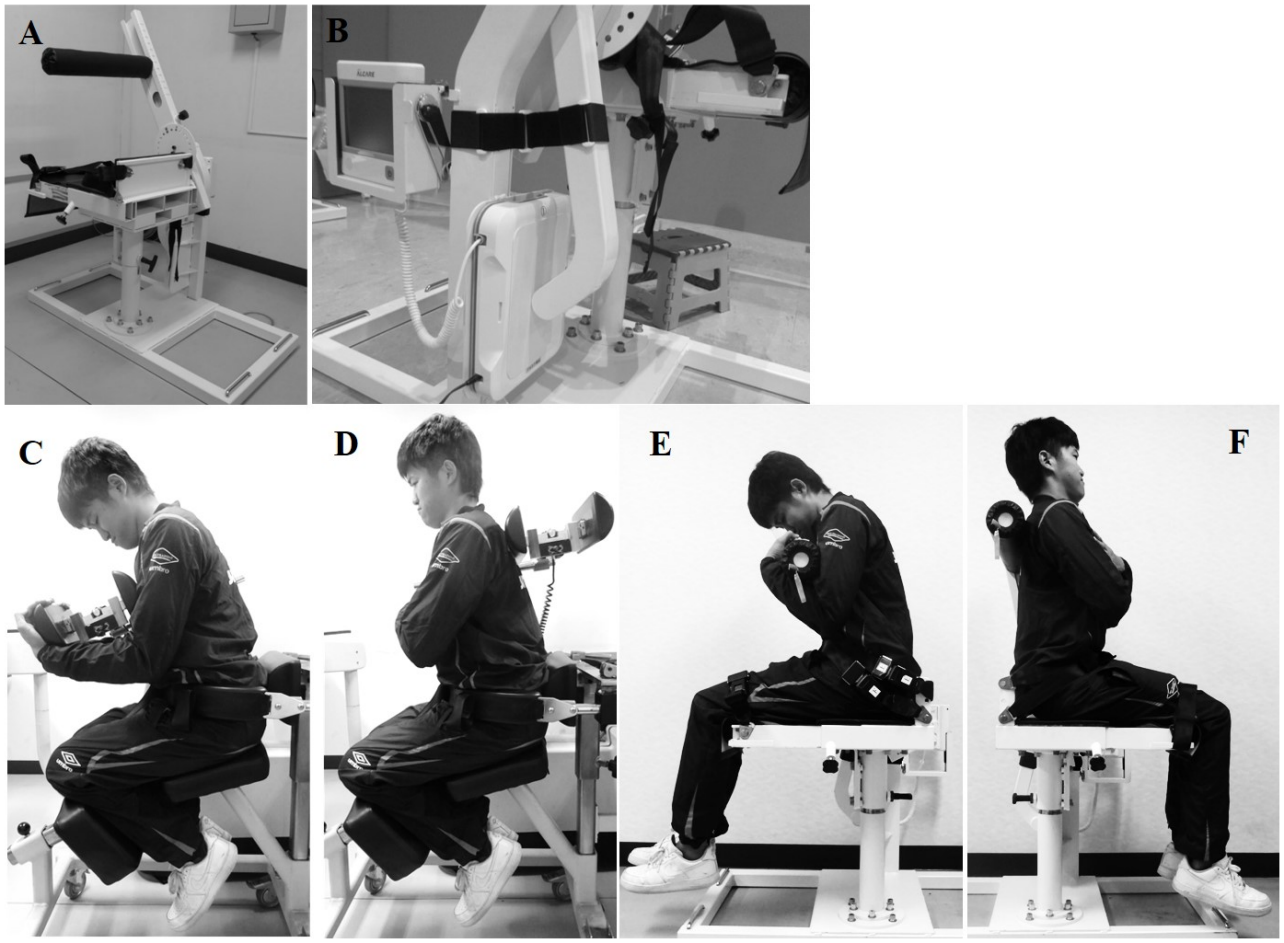
Portable trunk muscle strength measurement instrument. Overall appearance of the portable trunk muscle strength measurement instrument (A). The position on KinCom (B, C) and the portable trunk muscle strength measurement instrument (D, E).

In order to measure trunk muscle safely and easily, as well as in any location, we produced a novel portable trunk muscle torque measurement instrument (PTMI). The PTMI is loaded with a load cell for measurement, and consists of a simply fixable seat and torque stand (Fig 1A). We set a quadriceps torque measurement instrument (QTM)-06b with a load cell on the measurement portion. QTM-06b is a specifically adjusted version of QTM-05b, which is validated and available for measurement of quadriceps torque, and adapted for clinical evaluation and epidemiological studies [18,19]. The measurement error of QTM-06b was ± 10 N, and the maximum measurement range was 3000 N. Three adjustable belts were equipped with PTMI to fix subjects to the chair at the distal femur, proximal femur, and anterior superior iliac spine to prevent rotation of the pelvis and minimize the influence of the iliopsoas, quadriceps, and gluteus maximus. Just rotating the seat by 180 degrees with the subject seated, measurements of both flexion and extension torque could be attained quickly. Also, arm length, angle of the arm, and seat depth could be adjusted according to body size. The PTMI weighs 100 kg and is far more suitable for transportation compared with the 550-kg KinCom. In our practices, the PTMI was transported to the field while being carried by at least two adult men or women, and fit into a family car with its arm shortened. Here, it was necessary to examine the validity of the measurements with this new instrument. After validation, we applied this instrument to a large cohort study.

The first purpose of this study was to establish the validity of the newly produced PTMI. The second purpose was to estimate the reference data for trunk muscle strength in healthy young adults, and to reveal the development of age-related weakness. We hypothesized that by defining a reference for trunk muscle strength derived from young adults, it would contribute to a better understanding of the weakening that occurs with aging, and the influence of degenerative lumbar disease and trunk control in elderly people.

## Materials and methods

This report consisted of two studies. Study 1 was conducted to establish the validity and reliability of the PTMI. Study 2 was conducted to estimate a healthy young adult reference for trunk muscle torque and its relationship to aging. All participants in these studies provided written informed consent for the publication of these case details, and the study was conducted with the approval of the ethics committee of Hirosaki University.

### Study 1: Validity and reliability of a simplified portable trunk muscle measurement

#### Subjects

Twenty-four healthy students (16 males and 8 females) were recruited as volunteers from our university by phone calls and advertisements. They were recreational athletes and belonged to the soccer club or track and field club at their institution. There were no subjects who had low back pain.

#### Evaluation protocol

The trunk flexion and extension torque of all subjects were measured using two instruments. One measurement was with the KinCom 500H isokinetic dynamometer, which is a validated instrument [15]. Several novel instruments have been validated in previous articles by comparing the data with that acquired from the KinCom [20,21]. We also used the PTMI, which is described above. Both devices were used for the measurement of isometric torque. Subjects were classified into two groups; series of either KinCom to PTMI, or PTMI to KinCom. Measurements of flexion torque and extension torque were performed in alternate shifts to prevent experimenter bias. Before the measurement, all subjects performed five minutes of warm up that included systemic stretching. For use of the PTMI, subjects were positioned comfortably on the seat. The trunk was placed in 10° flexion, and the whole breast or back were fit parallel to the arm pad in order to minimize coupling with the axial rotational force. The height of the arm pad was adjusted for all individuals. For the flexion torque measurement, the center of the arm pad was positioned at the center of the sternum. The center of the arm pad was set at the level of the scapula for the extension torque measurement. All subjects were given the same instructions using an output monitor on how to measure the maximum torque before testing, and the same encouragement was provided during testing. Participants were allowed several submaximal practice efforts followed by sufficient rest. Only one maximal effort was recorded automatically by each instrument for torque (Nm) measurements. Strength testing was performed by orthopedic surgeons (S.S. and D.C.) trained and certified in both instruments. In our pilot study that included 20 young male athletes, the test–retest analysis indicated that the intraclass correlation coefficient (ICC (1,1)) of PTMI was 0.878 (p<0.001) for flexion torque and 0.834 (p<0.001) for extension torque.

#### Statistical analysis

The normal distribution of trunk extension and flexion torque data was confirmed using the Shapiro-Wilk test. We performed a post hoc power analysis using the standard deviations of the KinCom and PTMI data. Regarding trunk flexion in the 24 subjects, the standard deviations of PTMI and KinCom were 57.42 and 65.05, respectively, with a slope estimate of 0.6756 obtained when regression was performed for PTMI against KinCom. In order to reject the null hypothesis that the slope equaled zero to detect a 5% type I error, the power for trunk flexion torque was 0.999 to detect the 5% difference that was considered significant [22]. Also, using the same calculation, the power for trunk extension torque was estimated to be 1.000.

The flexion-to-extension (F/E) ratio was calculated from the flexion and extension torque. To estimate the validity and reliability of the flexion torque, extension torque, and F/E ratio, the interclass correlation coefficients (ICC (2,1)) were estimated. According to Landis’s criteria for consistency and reliability, the strength of agreement was considered poor when the correlation coefficient was below 0.00, slight when it was between 0.00 and 0.20, fair when it was between 0.21 and 0.40, moderate when it was between 0.41 and 0.60, substantial when it was between 0.61 and 0.80, and almost perfect when it was above 0.81 [23]. Furthermore, the root mean square (RMS) errors between the value of PTMI and KinCom were calculated to investigate the distribution of residual errors as follows:

RMSerror=√|KinComi−PTMIi|/(24).

### Study 2: Reference of trunk muscle torque and its relationship with aging

#### Subjects

Subjects were volunteers who participated in the Iwaki Health Promotion project. This project is a community-based program to improve expected lifespan through general health checkups and prophylactic interventions, and its details have been presented in previous reports [2,24–26]. All participants provided written informed consent, and the study was conducted with the approval of the ethics committee of our institution. Subjects aged over 18 years were recruited by calls from public health nurses and advertisements in the mass media. A total of 1054 volunteers (407 males and 647 females) from approximately 12,000 residents participated in 2013. Among them, 238 participants with self-reported low back pain who did not want to exert maximal effort, or those with a bone mineral density under 80% (considered low bone mass) of the young adult mean were excluded. Bone mineral densities were measured with a quantitative ultrasound measurement at the calcaneus (AOS-100NW, Hitachi Aloka Medical, Ltd., Tokyo). A total of 816 (317 males and 499 females) volunteers performed the measurement of trunk muscle torque.

#### Measurement of trunk muscle torque

Trunk muscle torque was measured using PTMI according to a standardized protocol. Full details of the torque testing protocol have been described in study 1. The maximum flexion and extension forces were recorded automatically, and each torque was calculated. Then, to control for the influence of body weight, torque per weight (Nm/kg) was estimated and used for statistical analysis. After the measurement, every subject was asked about the occurrence of injury or worsening low back pain. When an injury or a complaint about worsening low back pain occurred owing to a measurement, subjects had a physical examination and either an X-ray or magnetic resonance imaging of the injured site.

#### Statistical analysis

The reference interval for trunk muscle torque was estimated based on the mean and standard deviation values for healthy young adults aged 18–39 years (81 males and 105 females) and calculated by mean ± (2 × standard deviation) according to the general definition of sarcopenia [27,28]. Trunk sarcopenia was defined as a lower muscle torque than the lower limit of the reference interval. The prevalence of trunk sarcopenia was calculated among age groups: 40–49 (48 males and 73 females), 50–59 (49 males and 105 females), 60–69 (86 males and 132 females), 70–79 (46 males and 69 females), and 80 and older (80+) (7 males and 15 females)) in males and females. The age-related reduction was described as the torque percentage compared with the healthy young adult reference value. The values of trunk muscle torque among age groups were compared by one-way analysis of variance and the Tukey test. Data input and calculation were performed with SPSS ver. 12.0J (SPSS Inc., Chicago, IL, USA). A p-value of less than 0.05 was considered statistically significant.

## Results

### Study 1: Validity and reliability of PTMI

The mean age of the subjects in study 1 was 21.1 ± 2.5 (range 18–30) years, the mean height was 168.2 ± 7.9 (range 152–180) cm, and the mean body weight was 60.7 ± 8.0 (range 46–81) kg. No lumbar pain occurred in any subjects in study 1. The values of flexion torque measured using the KinCom were 214.4 ± 63.1 Nm in males and 141.4 ± 36.3 Nm in females (p = 0.006), and those of extension torque using the KinCom were 345.6 ± 74.3 Nm in males and 199.7 ± 49.6 Nm in females (p<0.001). The values of flexion torque measured using the PTMI were 209.5 ± 37.3 Nm in males and 120.9 ± 43.3 Nm in females (p<0.001), and those of extension torque using the PTMI were 450.6 ± 67.1 Nm in males and 242.8 ± 49.4 Nm in females (p<0.001). There were no significant gender differences in the F/E ratio between the KinCom (p = 0.120) and the PTMI (p = 0.610).

**Fig 2:**
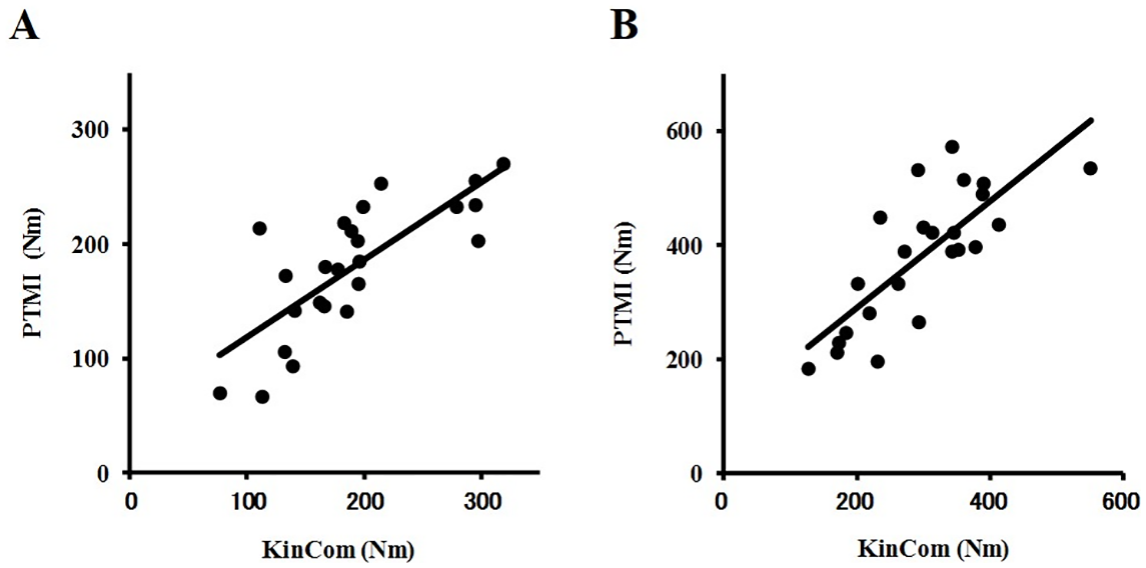
Scatter plot of flexion and extension torque by KinCom and the PTMI instrument. The flexion torque (A) and extension torque (B) were recorded using each instrument.

A validation study showed that the strength of agreement in flexion torque was almost perfect based on the ICC (2,1) being 0.807 (p<0.001) (Table 1) (Fig 2a). The strength of agreement for extension torque was substantial in that ICC (2,1) was 0.789 (p<0.001) (Fig 2b). The strength of agreement for the F/E ratio was moderate, with ICC (2,1) being 0.406 (p = 0.022). The values of flexion and extension torque using the PTMI were considered substantial to almost perfect in the validation based on the values acquired using KinCom. Also, calculated RMS errors were 3.2 Nm (1.7% of the mean value of KinCom) in flexion strength and 9.2 Nm (3.1% of the mean value of KinCom) in extension strength.

**Table 1:** Correlation coefficients for trunk muscle between by KinCom and PTMI in study 1.

	KinCom	PTMI	ICC(2,1)	p-value
Flexion torque (Nm)	190.1±65.1	180.0±57.4	0.807	<0.001
Extension torque (Nm)	297.0±96.4	381.4±117.0	0.789	<0.001
Flexion / Extension ratio	0.7±0.1	0.5±0.1	0.406	p = 0.022

Interclass correlation coefficients (ICC (2,1)) between KinCom and the portable trunk muscle strength measurement instrument (PTMI) were estimated.

### Study 2: Reference for trunk muscle torque and its relationship with aging

In study 2, the mean age of the participants was 53.2 ± 15.9 (range 18–89) years in males and 54.6 ± 15.0 (range 18–84) years in females, and there was no significant difference between genders. Based on the 10-day survey, one of 816 subjects (0.12%) had mild lumbar pain during the measurement, but the pain disappeared shortly without medical treatment. Also, there was no evident injury on any imaging studies.

**Fig 3:**
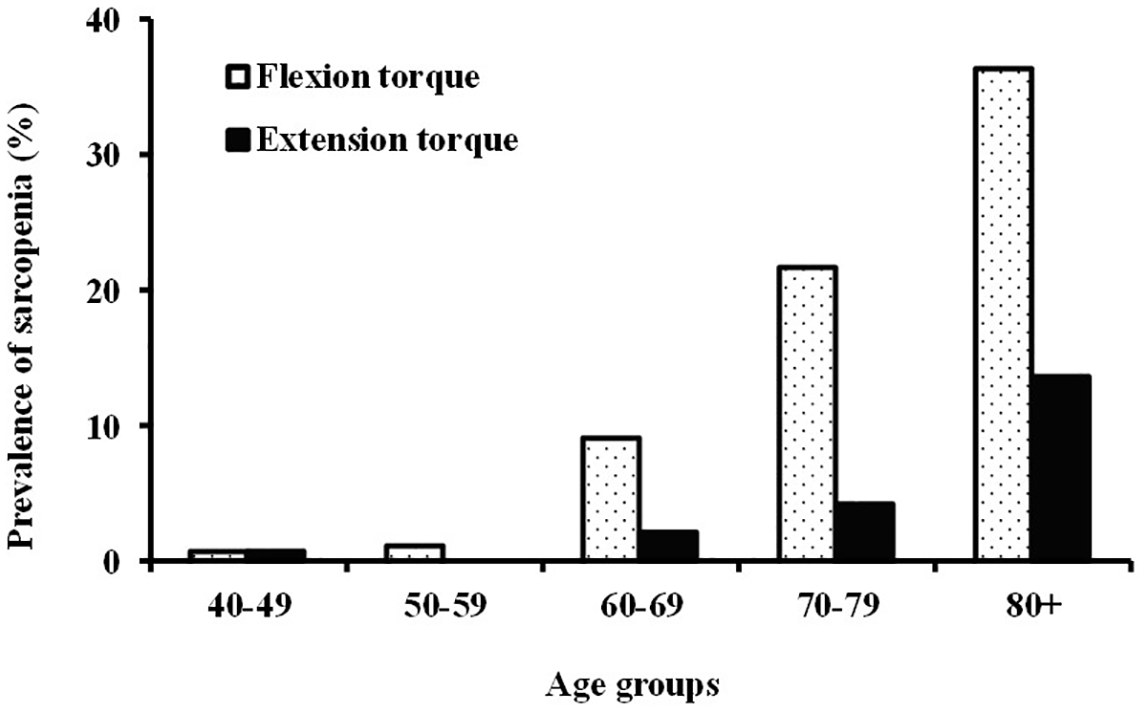
Prevalence of trunk sarcopenia. Values below the lower limit of reference intervals from young and healthy adults were defined as trunk sarcopenia. Subjects were divided into five groups: 40–49 (from 40 to 49 years of age), 50–59 (from 50 to 59 years of age), 60–69 (from 60 to 69 years of age), 70–79 (from 70 to 79 years of age), and 80+ (80 years of age and over).

Flexion and extension torque in males was 6.0 ±1.5 Nm/kg and 2.9 ±0.9 Nm/kg, respectively, and in females was 4.2 ±1.4 Nm/kg and 2.1 ±0.8 Nm/kg, respectively (Table 2). The young adult reference and the lower limit was derived from analysis of 96 young healthy males (Table 3). The young adult reference for flexion torque was 3.4 ±0.7 Nm/kg and the lower limit was 2.0 Nm/kg, while the reference for extension torque was 6.2 ±1.6 Nm/kg and the lower limit was 3.0 Nm/kg. In 113 young and healthy females, the young adult reference for flexion torque was 2.4 ±0.8 Nm/kg and the lower limit was 0.8 Nm/kg, while that of extension torque was 4.1 ±1.2 Nm/kg and the lower limit was 1.7 Nm/kg. The ratio of trunk sarcopenia under the lower limit in trunk muscle torque increased with aging, and a significant difference was observed after 60 years of age (Fig 3). Furthermore, the ratio of sarcopenia in flexion torque was higher than that of extension torque.

**Table 2:** Demographic data for participants in study 2.

	Males (n = 317)	Females (n = 499)	p-value
Age (y.o.)	53.2 ± 15.9	54.6 ± 15.0	0.296
Height (cm)	167.9 ± 6.9	155.2 ± 6.4	<0.001
Body weight (kg)	65.8 ± 10.0	53.3 ± 8.6	<0.001
BMI (kg/m^2^)	23.3 ± 3.0	22.1 ± 3.3	<0.001
Flexion torque (Nm/kg)	2.9 ± 0.9	2.1 ± 0.8	<0.001
Extension torque (Nm/kg)	6.0 ± 1.5	4.2 ± 1.4	<0.001
Flexion / Extension ratio	0.5 ± 0.2	0.5 ± 0.2	0.067

Age, height, body weight, body mass index (BMI), flexion torque, extension torque, and flexion / extension ratio were compared using Mann-Whitney U test. A p-value below 0.05 was considered significant.

**Table 3:** Mean value of trunk muscle strength of healthy young adults and the estimated reference interval.

	Males (n = 96)	Females (n = 113)
**Trunk flexion torque (Nm/kg)**		
Mean ± standard deviation	3.4 ± 0.7	2.4 ± 0.8
Reference interval	2.0–4.8	0.8–4.0
**Trunk extension torque (Nm/kg)**		
Mean ± standard deviation	6.2 ± 1.6	4.1 ± 1.2
Reference interval	3.0–9.4	1.7–6.5

Reference interval for trunk muscle torque was estimated based on the mean and standard deviation values of healthy young adults aged 18–39 years, and calculated as mean ± (2 × standard deviation).

**Fig 4:**
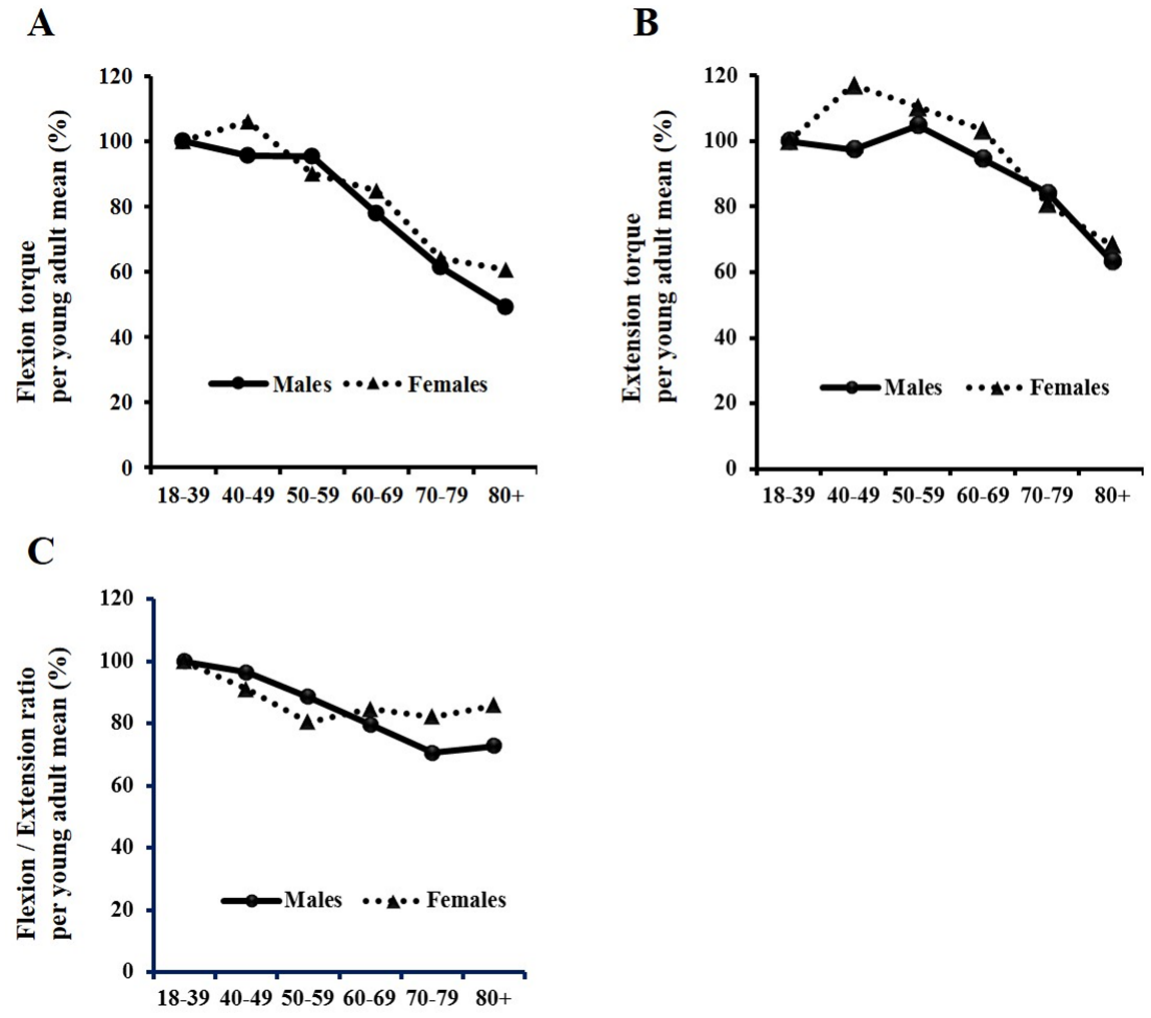
Age-related change in trunk flexion torque (A), extension torque (B), and flexion/extension ratio (C). The percentage of flexion and extension torque against the reference values for young and healthy adults are shown. Subjects were divided into four groups: <50 (under 50 years of age), 50–59 (from 50 to 59 years of age), 60–69 (from 60 to 69 years of age), and 70+ (above 70 years of age) in males and females.

Flexion torque compared with the young adult reference decreased significantly after 60 years of age and extension torque decreased after 70 years of age in males and females (Fig 4A–4C). In males over 80 years of age, trunk muscle torque compared to the young adult reference decreased to 49.1% in flexion and 63.5% in extension. In females over 80 years of age, trunk muscle torque compared to the young adult reference decreased to 60.7% in flexion and 68.4% in extension. The F/E ratio decreased significantly after 60 years in males and 50 years in females.

## Discussion

In this study, we created reference data for trunk muscle torque, and using a novel instrument validated in a large sample size, we revealed the age-related decrease in trunk muscle torque. These data were less affected by various diseases than in normal clinical studies because subjects were mainly healthy volunteers from the general population. The incidence of sarcopenia in trunk muscle increased significantly over the age of 60, and that of flexion torque was more than double that of extension torque. Furthermore, trunk muscle strength over the age of 80 decreased to approximately 60% of the young adult reference. These results clarified the age at which trunk muscle torque decreases, and suggested that there are many people at potential risk for low back pain, postural abnormalities, ambulatory disability, and poor outcomes after surgery.

In this study, a criterion-related validity test was adapted, and the validity of PTMI for KinCom was supported by high values of ICC (2,1). It has been shown that values between 0.61 and 0.80 have moderate, and those between 0.81 and 1.00 have a strong correlation between two parameters [23,29]. According to these cut-offs, trunk flexion and extension torque using PTMI had a strong correlation with KinCom. Also, the mean strength values (Nm) evaluated in study 1 were similar values to concentric force at 10 degrees per second compared with 40 degrees per second measured by KinCom in a younger population [30]. This information supported the accuracy of our measurement method, as this condition for isokinetic strength measurements was considered to be similar to the conditions for isometric measurement. However, we must take into account the fatigue and learning effect in measuring trunk muscle torque as well as general torque measurements [31]. There was no significant learning effect in 3 trials performed at 2-day intervals if sufficient instructions were given and the output monitor was checked before the measurements. Also, remarkable fatigue was not apparent in 3 trials per day performed at 5-minute intervals. Sufficient rest and instructions on how to create maximum torque may prevent fatigue and learning effects. On the other hand, we adopted the isometric measurement for the purpose of safety, while the dynamic torque of the trunk has typically been measured with sophisticated dynamometers that control motion while measuring the force being exerted. It is known that an individual’s strength is reduced by 10±30% when exertions are performed dynamically as compared with isometrically, which means that given the same exertion level, a dynamic exertion would be closer to the strength capacity of the muscle compared with a static exertion, resulting in a higher risk of muscular injury [32–34].

The European working group on sarcopenia in older people reported a consensus on the definition and diagnosis based on low muscle mass plus low muscle strength or low physical performance [35]. This international definition is broadly used in many countries, including Japan [36]. Although there was no mention of trunk muscle mass or strength in this report, they generally recommended the use of normative (healthy young adult) rather than other predictive reference populations, with cut-off points at two standard deviations below the mean reference value. Our criteria were different in that we did not identify low muscle mass because the bioelectrical impedance analysis identified so few subjects with sarcopenia based on whole body muscle mass. However, it clearly showed that the prevalence of sarcopenia increased with aging based on strength measurements. There are few previous studies that have considered or adjusted for age-related reductions of strength in the trunk muscles. Frontera et al. reported from their small sample in a 12-year longitudinal study that skeletal muscle isokinetic strength decreased 0.75–2.45% per year, and mid-thigh muscle cross-sectional area decreased 1.0–1.3% per year in men around 65 years of age at baseline [37]. Furthermore, 0.5–0.6% muscle loss occurred with just 10 days of bed rest [38–40]. Abe et al. reported that sonographic muscular thickness achieved a peak at 50 years of age, then continued to reduce gradually [41,42]. They also showed that the reduction of muscle thickness began at age 50, and the prevalence of sarcopenia at age 60 was only 10% in males and 21% in females. This suggested that there is a discrepancy between muscular volume and muscular recruitment, and it is not accurate to estimate physical function based on muscular volume or thickness alone. One of the possible explanations for the discrepancy with our results could be differences in neuromuscular control. Age-related alterations in the neuromuscular system have recently been shown to be the result of functional denervation [43,44].

Many other factors may be responsible for the reduction in trunk muscle that begins around the age of 60. Although trunk muscle strength differs between males and females because of the high prevalence of lumbar spondylosis in males [26], there were no remarkable differences in age-related reduction patterns; that is, the flexion torque and extension torque per weight decreased to 60% of the healthy young adult reference. On the other hand, Imagama reported that back muscle weakness was correlated with lumbar kyphosis and the disc degeneration score, and their multiple regression analysis revealed that weakness most strongly affected the physical component summary in SF-36 [45]. Further analysis is needed to explain why trunk muscle torque decreases to approximately 60% of the young adult reference, and its influence on body stabilization and spinal alignment.

While low trunk extension strength is associated with poor surgical outcomes, trunk strength is not the sole factor contributing to this. Many studies have concluded that maintaining trunk extension strength is necessary to achieve good postoperative outcomes and prevent low back pain [16,46–49]. Patients with poor outcomes had lower back muscle strength. The contributing factors could include disuse atrophy caused by longstanding pain, a sudden change of sagittal spinal alignment, or denervation due to excessive intraoperative retraction. In practice, the importance of trunk flexion torque is not fully understood. These results revealed that the decrease of trunk flexion torque began at a younger age than extension torque by focusing on the relationship with aging, and there have been a few reports that have shown the importance of the imbalance of trunk flexion and extension strength. McGills suggested that it is important to focus not only on extension strength but also flexion strength, owing to the importance of antagonistic muscle activity [50]. Kim et al. reported that lordosis is related to the F/E ratio [5]. In this study, there was no significant difference in the balance of trunk flexion and extension torque between males and females. The F/E ratio changed 0.41–0.56 in males and 0.50–0.56 in females. It is controversial which value is appropriate for trunk stabilization, but it must be considered with spinal alignment carefully in future analyses.

There were several limitations of this study. First, there was a disproportionate ratio of females to males. Most of the analyses were conducted separately in males and females, but this may have had a slight influence on the variability or accuracy of the mean values. Second, from the pilot validity study, we could not predict the safety of the device for use in elderly patients with osteoporosis, degenerative spondylosis, or chronic low back pain. However, there were no severe side effects such as vertebral fractures or worsening pain during the large sample cohort study, when we excluded only those with severe osteoporosis and those who did not want to exert maximal effort because of preexisting low back pain. These exclusion criteria for safety may have led to selection bias, possibly resulting in less accurate measurements in the elderly subjects. However, we considered that there were no significant effects on the young adult reference. On the other hand, more severe criteria would be necessary to estimate a pure normative range of trunk muscle by removing the degenerative alignment or pain factors, because these factors affect the trunk torque and reduce the mean values among each age-sex group. Evaluation of spinal alignment using imaging or physical examination was not performed. Although it is challenging to fully estimate the presence or degree of pain using imaging alone, trunk muscle strength, tension, and laxity would be affected by sagittal spinal alignment. While this point is important and interesting, it was not realistic to perform lateral whole-spine radiographs in this large number of subjects. We are planning to overcome this point and reveal the relationship between trunk muscle strength, postural imbalance (i.e. occiput wall distance), spinal alignment imaging, range of motion, and symptoms (including pain) in a future study. Also, our results may not be fully generalizable because we selected a fairly homogeneous group of university students who play on sports teams. While a broader and more heterogeneous population would be more suitable for statistical analysis owing to trunk torque values being more variable, we conducted reliability analysis for PTMI with a power of 0.99–1.00. It is also necessary to consider the limited stabilization afforded by the PTMI. Pursuing a lighter and more portable device would lead to the compromise of this device. Finally, we had to consider that quantifying torque generation in flexion and extension is not easily achieved. Physical, mental, and methodological factors can affect the exertion of maximal effort. To overcome these challenges, we attempted to create consistent protocols regarding the subjects’ setting, exertion, and coaching. Furthermore, to reduce the variation of data due to individual differences, an investigation of normative values was conducted in a large sample cohort.

Despite these limitations, this large sample cohort study included subjects with all varieties of jobs, lifestyles, physical function levels, and lumbar symptoms, allowing the development of reference data for trunk muscle strength. We hope that the relationship between degenerative spinal disease, symptoms, physical function, imaging studies, and trunk muscle strength will be investigated in future studies based on these reference data.

## Conclusion

The validity of PTMI was ensured by correlating the results obtained with it to those obtained with KinCom. PTMI results led to the establishment of reference data for healthy young adults and demonstrated an age-related reduction in trunk muscle torque. Trunk sarcopenia progressed with age, and loss of flexion torque began earlier than loss of extension torque. These results contribute to a better understanding of trunk stability and may lead to the development of potential preventive interventions.
